# SENIOR TECHNOLOGY ACCEPTANCE, FEAR OF FALLING AND FALL RISK ASSESSMENT IN COMMUNITY-DWELLING OLDER ADULTS

**DOI:** 10.1093/geroni/igad104.3585

**Published:** 2023-12-21

**Authors:** Eunice Oladepe Ojo, Boon Peng Ng, Ladda Thiamwong

**Affiliations:** University of Central Florida, Orlando, Florida, United States; University of Central Florida, Orlando, Florida, United States; University of Central Florida, Orlando, Florida, United States

## Abstract

The use of technology, such as the BTrack balance system (BBT) for fall risk assessment and the Senior Technology Acceptance (STA) survey is important for understanding technology-based fall prevention efforts for at-risk older adults. The purpose of this study was to examine the relationship between technology-based fall risk assessment and STA among community-dwelling older adults aged 60 years and older (n=124). Descriptive statistics, bivariate analysis, and multiple linear regression adjusted for socio-demographics were performed. The STA tool was used to obtain information about older adults’ acceptance of technology. The STA consists of four domains and 14 items. The Short Fall Efficacy Scale International (FES-I) and the BBT were used to measure fear of falling and balance performance, respectively. Of the study population, 77.4% were female, 22.6% were non-white, 8% reported financially inadequate, 46% had low BBT scores and low FES-I scores, whereas 33.9% had high BBT and high FES-I scores, and 20.2% had low BBT and high FES-I scores. For the bivariate analysis, we found a statistically significant difference in the STA scores based on age (p=0.006) and education (p=0.032). In the adjusted analysis, only age was negatively associated with the STA (p=0.004); fall risk assessment using the BBT and FES-I were not significantly associated with STA. With a small study population, readers should be aware of this limitation to our findings. Further studies are needed to address this issue and to identify effective strategies to encourage older adults to use technology to improve their well-being.

